# Early intermittent cholesterol exposure accelerates atherosclerosis: an oscillating amplifier calling for preemptive control

**DOI:** 10.1038/s41392-024-02032-7

**Published:** 2024-11-07

**Authors:** Maria Kral, Yvonne Döring, Christian Weber

**Affiliations:** 1 Institute for Cardiovascular Prevention (IPEK), Ludwig-Maximilians-Universität München (LMU Munich), Pettenkoferstr 9, 80336 Munich, Germany; 2 DZHK (German Center for Cardiovascular Research), Partner Site Munich Heart Alliance, Munich, Germany; 3 Department of Angiology, Swiss Cardiovascular Center, Inselspital, Bern University Hospital, University of Bern, Bern, Switzerland; 4 Department of Biochemistry, Cardiovascular Research Institute Maastricht (CARIM), Maastricht University Medical Centre, Maastricht, The Netherlands

**Keywords:** Innate immune cells, Cardiology

A recent publication in *Nature*^1^ identified early intermittent hyperlipidemia as a new determinant of atherosclerosis driving an altered phenotype of arterial-resident macrophages with defects in autophagy- and efferocytosis-related pathways. These intriguing findings infer the need for preemptive control of cholesterol already at younger age to prevent and limit atherosclerosis.

**Fig. 1:**
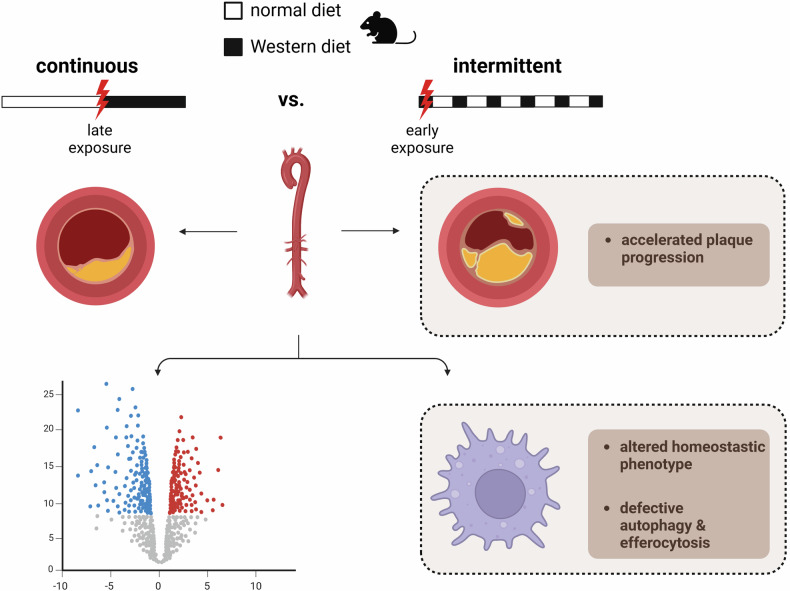
Early exposure and subsequent intermittent diet of a cholesterol-rich Western diet (21% fat, 0.15% cholesterol) leads to accelerated plaque development as compared to a continuous Western diet. Mice received a total of 6 weeks of Western diet in both diet groups. Mice in the intermittent fasting group received cycles of a Western diet for 1 week, followed by two weeks of normal diet. RNA sequencing revealed of arterial-resident macrophages revealed an altered homeostatic phenotype concomitant with defects in autophagy- and efferocytosis-related pathways. Made with biorender.com

More specifically, to assess early cholesterol exposure, Takaoka et al. initiated dietary intervention at 6 weeks of age as compared to “traditional” models where hyperlipidemia is induced after 8 weeks. Intriguingly, they found that compared to *LDLR*^*−/−*^ mice fed a continuous Western diet (WD), early intermittent hyperlipidemia significantly accelerated atherosclerotic plaque development in both sexes (Fig. 1). To unravel mechanistic cues underlying the effect of early intermittent hyperlipidemia, they performed RNA-sequencing of plaque-bearing aortae in male *LDLR*^*−/−*^ mice fed either continuous (cWD) or intermittent WD (iWD) for 3 weeks, with a particular focus on macrophages. Remarkably, the analysis revealed an altered homeostatic phenotype of resident-like arterial macrophages, which failed to acquire functions rendering them atheroprotective upon early intermittent hyperlipidemia. This finding is in line with another recent publication describing an acceleration of atherosclerosis in *LDLR*^*−/−*^ mice fed an early alternating high-fat diet as compared to a late continuous HFD for 8 weeks.^2^ More specifically, RNA sequencing of the aorta in these mice revealed an alteration of neutrophil reprogramming upon early exposure and alternating HFD, as compared to a continuous HFD. Both studies highlight the detrimental consequences of an early exposure of high levels of circulating lipids and should be implemented in the design of more effective short-term drugs and prevention strategies for the treatment of ASCVDs.

Furthermore, the RNA-sequencing data by Takaoka et al. indicated impairments in pathways of autophagy and efferocytosis in arterial macrophages with several changes in the expression of genes related to autophagy (e.g. *Atg12*, *Vps33a, Wdfy4*,), which were reduced in the aorta of male iWD-fed *Ldlr*^*−/−*^ mice (Fig. 1). This reduction was linked to enhanced apoptotic debris and an enlarged necrotic core in the lesions. Another interesting observation was the strong reduction of lymphatic vessel endothelial hyaluronic acid receptor 1 (LYVE1^+^) macrophages, a subset previously described as aortic resident population.^3^ In *ApoE*^*−/−*^ mice fed a WD, the authors found a progressive reduction of LYVE1^+^ macrophages over time, which negatively correlated with atherosclerotic plaque burden. Importantly, resident-like LYVE1^+^ macrophages were also reduced in tissue sections of human atherosclerotic coronary arteries from patients with coronary artery disease as compared to healthy arteries. To further investigate the role of this macrophage subset, the authors generated *ApoE*^*−/−*^ mice that lacked LYVE1^+^ expression in macrophages (*Lyve1*^*Cre+/wt*^*Csf1r*^*flox/flox*^*)*. When fed a cWD for 20 weeks, plaque size was significantly increased. The impact of 6 weeks cWD versus iWD was also assessed in a different model of hyperlipidemia after injecting AAV8-D377Y-mPCSK9 in *Lyve1*^*Cre+/wt*^*Csf1r*^*flox/flox*^ mice. Interestingly, iWD-fed mice lacking LYVE1^+^ macrophages did not display increased plaque size, indicating that this subset of macrophages becomes altered in the course of atherosclerosis progression, thereby accelerating disease progression. Thus, the potential of targeting this arterial resident macrophage subset for future clinical interventions clearly merits further investigation, especially as intermittent situational treatment of cholesterol exposure is challenging. However, it would be also crucial to understand how iWD shapes the role of pro-inflammatory macrophages in the development of atherosclerosis.

As putative targets, genes related to “actin filament organization” were differentially expressed in resident macrophages of cWD- compared to iWD-fed *LDLR*^*−/−*^ mice. For example, *NRP1* was downregulated in aortic macrophages of iWD-fed *LDLR*^*−/−*^ mice. To dissect the role of NRP1 in resident-like macrophages, they transplanted bone marrow of mice that lacked NRP1 in myeloid cells (*Lyz2*^*Cre+/*−^*Nrp1*^*flox/flox*^) into *LDLR*^*−/−*^ mice. Interestingly, while bone marrow reconstitution of *LDLR*^*−/−*^ mice fed a cWD led to an increase in plaque area, this effect was not seen in iWD-fed mice. These data suggest that early exposure to high levels of circulating lipids alters the protective functions of NRP1 in resident-like macrophages in rodents.

Hyperlipidemia has emerged as a major risk factor for the development of atherosclerotic cardiovascular disease (ASCVDs) defined by lipid- and immune cell-rich deposits in the arterial wall.^4^ Sustained inflammation and lipid deposition lead to plaque growth and eventually rupture, causing thrombosis, which can clinically manifest as myocardial infarction or stroke. Given that atherosclerosis is associated with age, current risk stratification and therapeutic strategies to lower LDL levels are mostly applied in adulthood or after events. However, a growing body of evidence suggests that early exposure to cholesterol, especially with periodic variations, is involved in the pathogenesis of atherosclerosis.^4^

In this regard, Takaoka et al. analyzed data from the Cardiovascular Risk in Young Finns Study (YFS), which was conducted in 1980 to assess determinants of cardiovascular risk factors in young participants.^5^ This analysis confirmed that cholesterol exposure early in life can associate with increased carotid atherosclerotic plaques in mid-life.

Overall, this study reveals for the first time the impact of an early intermittent hyperlipidemia on atherosclerosis development. The group led by Ziad Mallat could establish how such early intermittent exposure to high circulating lipids modulates crucial biological pathways in resident-like arterial macrophages, thereby affecting their homeostatic phenotype. They further found impaired autophagy- and efferocytosis-related pathways in arterial macrophages, which accelerated atherosclerosis. However, in the present study they focused on effects after short exposure of 6 weeks to early intermittent WD. It would be interesting to follow up on their findings by expanding the duration of iWD and cWD for comparison with continuous WD or normal diet. Moreover, it would be pivotal to understand how extended periods of a normal diet in-between cholesterol-rich episodes or changes in their frequency affect atherosclerosis progression or can even promote regression. Finally, future studies should also address the effects of this specific diet regimen at different age ranges. The association of an early intermittent hyperlipidemia with cardiovascular events in human adulthood harbors important implications. With respect to lipid-lowering treatment, discontinuation of statins revealed an association with increased cardiovascular event rates.^4^ Therefore, their observations should be taken into consideration for short-term treatment interventions (instead of cholesterol deprivation) at earlier time points as well as with regards to the continuity of statin treatment. Moreover, autophagy induction might be exploited as a potential strategy by targeting autophagy-related genes altered upon early intermittent hyperlipidemia. Alternatively, or additionally, chronic modulation of LYVE^+^ macrophages to restore their atheroprotective functions could be developed as novel anti-atherogenic therapeutic strategy.

Together, the study by Takaoka et al. identified early intermittent exposure to cholesterol as a novel driver fueling atherosclerosis, like an oscillating amplifier compromising music, and thereby highlights the importance of consequently and preemptively maintaining low levels of cholesterol already at a young age.
